# Factors Contributing to Geographical Variation in Maternal Smoking Rates Among Aboriginal and Torres Strait Islander Women

**DOI:** 10.1002/hpja.70095

**Published:** 2025-09-07

**Authors:** Emilie Cameron, Matthew Clapham, Rita Hitching, Sandra Eades, Bob Davis, Jennifer Rumbel, Kristy Fakes, Jamie Bryant

**Affiliations:** ^1^ School of Medicine and Public Health, College of Health, Medicine and Wellbeing University of Newcastle Callaghan New South Wales Australia; ^2^ Equity Health and Wellbeing Program Hunter Medical Research Institute New Lambton Heights New South Wales Australia; ^3^ Clinical Research Design and Statistics Support Unit Hunter Medical Research Institute New Lambton Heights New South Wales Australia; ^4^ Centre for Epidemiology and Biostatistics, School of Population and Global Health University of Melbourne Carlton Victoria Australia; ^5^ Systems Neuroscience Group, School of Psychological Sciences Hunter Medical Research Institute New Lambton Heights New South Wales Australia

**Keywords:** Australian Aboriginal and Torres Strait Islander peoples, indigenous peoples, maternal health, socioeconomic factors, tobacco smoking

## Abstract

**Issue Addressed:**

Smoking during pregnancy poses serious health risks for mother and baby. Addressing smoking among pregnant Aboriginal and Torres Strait Islander women is an Australian national priority. This study aimed to understand the geographical variation in rates of not smoking during pregnancy among Aboriginal and Torres Strait Islander women.

**Methods:**

Data from the National Perinatal Data Collection were obtained for all births in Australia recorded between 2014 and 2017 to women aged 18 and over who were recorded as Aboriginal and/or Torres Strait Islander. Sociodemographic characteristics were obtained from national data for each of the 340 included geographic areas of residence (SA3). The characteristics associated with not smoking in the first 20 weeks of pregnancy were explored with conditional autoregressive spatial regression modelling.

**Results:**

Over half (56%) of the 49 341 women included in the dataset reported they did not smoke in the first 20 weeks of pregnancy. The prevalence of not smoking ranged from 39% to 86% across geographic areas. Not smoking was highest in areas with higher median age, lower levels of socio‐economic disadvantage and increased participation in employment.

**Conclusions:**

Not smoking during the first 20 weeks of pregnancy among Aboriginal and Torres Strait Islander women was strongly associated with area‐level socioeconomic disadvantage driven primarily by the level of employment in the area.

**So What?:**

Targeted public health strategies that focus on areas identified as having high rates of maternal smoking and on improving employment opportunities and addressing socioeconomic disadvantage could contribute to a reduction in smoking rates.

## 1 Introduction

Smoking during pregnancy is the most significant modifiable cause of adverse pregnancy outcomes for both mother and child [1].Maternal smoking is a significant risk factor for pre‐term and low birth weight and neonatal mortality [2, 3, 4]. Other impacts are lifelong and include increased risk of neurological and developmental disorders including attention‐deficit/hyperactivity disorder and learning disabilities, asthma, and an increased likelihood of smoking initiation in adulthood [2, 5].

In Australia, the proportion of Aboriginal and Torres Strait Islander women reporting not smoking during pregnancy has increased over the last decade, from 49.3% in 2011 to 58.7% in 2022 [6]. Although this progress is encouraging, continued efforts are needed to further support smoke‐free pregnancies and improve maternal and child health outcomes. As such, reducing smoking among Aboriginal and Torres Strait Islander women remains a national priority under the Closing the Gap agreements [7].

In Australia and globally, Indigenous populations are disproportionately represented among populations with higher smoking prevalence and are more likely to be impacted by the social and structural determinants of smoking [8]. These factors include employment, education, access to health care and support, psychological distress and discrimination [9]. High rates of smoking are directly linked to colonisation, forced assimilation and systemic inequalities, and the targeted introduction of tobacco into Indigenous communities [10].

Research has shown that most Aboriginal and Torres Strait Islander people who smoke want to quit and there are efforts underway to develop culturally safe, acceptable and effective strategies to address smoking [11]. Although there is limited evidence for tobacco control strategies that are effective with Aboriginal and Torres Strait Islander women [12] there are some strategies that show promise including counselling and support delivered by an Aboriginal woman or Aboriginal Health Worker, including the woman's partner and other adults in supporting quit attempts and the provision of free nicotine replacement therapy. These strategies have been shown to be acceptable and feasible in a range of settings [13, 14, 15]. In addition, there is ongoing work examining the effectiveness of group‐based cessation approaches delivered in Aboriginal Community Controlled Health Services [16, 17].

Data provided in the Child and Maternal Health web report [18] found that during 2014–2016, rates of not smoking during pregnancy by Aboriginal and Torres Strait Islander women varied across local geographical areas, ranging from 44% in North West Victoria (VIC) to 73% in Gold Coast, Queensland (QLD). Australia's vast and diverse landscape creates unique challenges for healthcare access during pregnancy and disproportionately impacts rural and remote communities [19, 20]. There is a need for further exploration of differences in smoking by geographical location, and what might be driving these differences to inform the targeted provision of smoking prevention and cessation services. To address this gap, the current study aims to examine the sociodemographic factors associated with geographical variation in rates of not smoking during pregnancy among Aboriginal and Torres Strait Islander women and identify areas of high and low rates of not smoking.

In line with the Lowitja Institute's guidance on strengths‐based reporting [21], this study adopted a strengths‐based approach that goes beyond simply reversing deficit‐based narratives, instead actively centring the strengths, culture, diversity and rights of Aboriginal and Torres Strait Islander people in both framing and interpretation.

## 2 Methods

### 2.1 Aboriginal and Torres Strait Islander Governance and Research Team Positionality

We recognise that the lived experience and worldviews of the research team influence the design and interpretation of the study. SE (Noongar) and JB (non‐Indigenous) conceptualised the study as part of a National Health and Medical Research Centre for Research Excellence Grant focused on Aboriginal Child and Adolescent Health. The research team comprised Aboriginal and Torres Strait Islander researchers with lived experience (SE, BD (Dungutti/Biripi), JR (Gamilaraay)), specialists in Aboriginal health services research (SE, BD, JR, JB), health behaviour scientists (JB, EC, KF, RH) and data scientists (MC, EC). An Aboriginal and Torres Strait Islander Reference Group was established to oversee the research process, provide advice on the study design and support the interpretation of findings. The group ensured the research was conducted in accordance with core values for ethical research with Aboriginal and Torres Strait Islander peoples including spirit and integrity, reciprocity, respect, equity, responsibility and cultural continuity [22]. The group consisted of five Aboriginal members (SE, BD, JR, Nicole Turner (Kamilaroi) and Steve Blunden (Dunghutti)) with expertise in Aboriginal health research, policy development and evaluation and health care delivery.

### 2.2 Ethics Approvals

Ethics approvals were obtained from the Aboriginal Health and Medical Research Council (1588/19), Aboriginal Health Research Ethics Committee SA (04‐19‐853), ACT Health Human Research Ethics Committee (2019.ETH.00231), Human Research Ethics Committee of Northern Territory Health and Menzies School of Health Research (2019‐3580), QLD Public Health Act (EO2020/1/1134), SA Health HREC, University of Newcastle’s Human Research Ethics Committee (H‐2019‐0414), and the Australian Institute of Health and Welfare Ethics Committee (EO2020/1/1134). The research was conducted in line with the National Health and Medical Research Council's Guidelines for ethical conduct in Aboriginal and Torres Strait Islander health research [22], the Aboriginal Health and Medical Research Council's ethical guidelines: key principles (2020) V2.0 [23] and the international CONSIDER statement [24].

### 2.3 Study Design

Secondary analysis of data from the National Perinatal Data Collection (NPDC) for the period 2014–2017.

### 2.4 Data Source

The NPDC is a large, comprehensive set of data on pregnancy and childbirth in Australia that is collected using a population‐based surveillance system. The cross‐sectional dataset consists of an agreed set of standardised data items, as specified in the Perinatal National Minimum Dataset, as well as additional items. For every birth, notification forms are filled out by midwives and other staff using information obtained from mothers and from hospital, antenatal or other records. This data is supplied to the Australian Institute of Health and Welfare (AIHW) to form the NPDC [25]. For this study, extracted NPDC data was accessed using the Sax Institute Secure Unified Research Environment (SURE) from March 2022.

### 2.5 Study Population

Data was provided by the NPDC for all births recorded between 1st January 2014 and 31st December 2017 to women aged 18 and over who were recorded as Aboriginal and Torres Strait Islander. Only records that had smoking status in the first 20 weeks of pregnancy recorded were included in the analysis.

### 2.6 Scale

The Statistical Area (SA) structure of the Australian Statistical Geography Standard developed by the Australian Bureau of Statistics [26] for the collection and dissemination of geographic statistics was selected as the most relevant geographic structure for this work. The SA structure is hierarchical, where lower‐level units fit wholly into higher‐level units. For this study, data was obtained by SA3. The SA3 areas provide a standardised regional breakup of Australia focusing on creating functional regional areas. There were 340 SA3 areas represented in the dataset, each with a population of 30 000–130 000 people. Our sample contained SA3 versions from 2011 and 2016. Any area that was split into separate statistical areas in 2016 was recombined to be consistent with the 2011 version, with characteristics combined using a population weighted method.

### 2.7 Measures

#### 2.7.1 Smoking Status

The main outcome for this study was self‐reported not smoking during the first 20 weeks of pregnancy. Women were classified as not smoking if did not smoke was recorded for the NPDC variable ‘Did you smoke at all in the first 20 weeks of pregnancy’ [27].

#### 2.7.2 Geographic Area

The SA3 of usual residence was provided for each birth in the NPDC data extraction. Area of usual residence is derived from the woman's address, defined as the place where the person has or intends to live for 6 months or more; the place that the person regards as their main residence; or where the person has no other residence, the place they currently reside [28].

#### 2.7.3 Area Level Factors

Australian Bureau of Statistics regional data [29] was used to obtain sociodemographic variables for each SA3 including: total population size and median age (from 2015 population and people data cube); median weekly income (from 2015 Income data cube); proportion of population participating in paid employment and proportion who completed year 12 or equivalent (from 2016 education and employment data cube); and proportion aged over 18 with private health insurance (from 2015 health and disability data cube). Remoteness, based on the Accessibility and Remoteness Index of Australia (ARIA) from the 2011 Australian Statistical Geography Standard, and area level socioeconomic disadvantage quintiles from the Australian Bureau of Statistics 2011 Socio‐Economic Index for Areas (SEIFA) Index of Relative Socio‐economic Disadvantage (IRSD) were obtained for each SA3.

### 2.8 Analysis

All statistical analysis were programmed using R version 4.0.3 (2020‐10‐10) [30]. The characteristics of the SA3 of usual residence for each woman in the dataset were stratified by smoking status and summarised with median and range (min and max) or frequencies and percentages as appropriate.

Spatial conditional autoregressive (CAR) regression modelling was used to assess the association between SA3 area‐level factors and not smoking during pregnancy. The CAR regression model accounts for spatial correlations through a spatially correlated random effect. Within the CAR model, the correlations among SA3s are scaled by the estimate ρ (rho); higher positive values mean not smoking is more similar on average to neighbours compared to non‐neighbours. Moran's I, another measure of spatial autocorrelation, was calculated on the proportion of not smoking within each SA3. Using SA3 Australian Statistical Geography Standard digital boundary files provided by the Australian Bureau of Statistics [26] with the R package spdep [31], a neighbourhood weight matrix with 1 representing that 2 areas are neighbours and 0 otherwise was constructed to account for correlations.

There was evidence of multicollinearity between the factors SEIFA, income, employment, and education. This was not surprising given that SEIFA is a composite measure incorporating income, education, employment and housing variables [32]. Two multivariate CAR spatial regressions were therefore constructed: Regression (1) included the factors—age, total population size, private health insurance, remoteness and SEIFA, and excluding income, employment and education; and Regression (2) included the factors—age, total population size, private health insurance, remoteness, income, employment and education, and excluding SEIFA. For the regressions, total population size was scaled to estimate the change in odds due to a 10 000 increase in population and income scaled to estimate the change in odds due to a $100 increase in weekly income. Results from crude regressions and the two multivariable CAR spatial regressions are presented. Results for the fixed effects parameters are provided as odds ratios with confidence intervals and for the random effects parameters as rho (*ρ*). *p*‐values were calculated from likelihood ratio tests and a bootstrap likelihood ratio test was conducted to assess the significance of the spatial correlation parameter. Confidence intervals were calculated using the profile likelihood method.

A heatmap of Australia was generated using the CAR spatial regression model, incorporating SEIFA while excluding income, employment and education. For the map, SA3s with less than 10 women (or 300 women for NT SA3s) were merged with adjacent SA3 areas and the model recalibrated in order to retain the privacy of the women. The areas with the highest and lowest rates of not smoking were identified.

## 3 Results

### 3.1 Sample

There was a total of 49 651 births to Aboriginal or Torres Strait Islander women aged 18 and over in Australia between 2014 and 2017 with smoking status in the first 20 weeks of pregnancy recorded. Of these, 310 had a special purpose or no usual address SA3 code and were excluded. Table 1 presents sample characteristics at the SA3 level stratified by smoking status. Overall, 28% (*n* = 13 659) of the sample lived in an area classified as outer regional and 27% (*n* = 13 096) in a major city. Forty percent (*n* = 19 853) lived in an area classified as most disadvantage compared to 4% (*n* = 1845) in an area of least disadvantage. Over half (*n* = 27 607, 56%) reported they did not smoke during the first 20 weeks of pregnancy.

**TABLE 1:** SA3 level descriptive statistics of the usual residence of pregnant Aboriginal and Torres Strait Islander women stratified by smoking status (*N* = 49 341).

Characteristic	Did not smoke^a^	Smoked^b^
	*N* (%)	*N* (%)
Total	*N* = 27 607 (56%)	*N* = 21 734 (44%)
Remoteness (ARIA)		
Major Cities of Australia	7966 (61%)	5130 (39%)
Inner Regional Australia	5466 (59%)	3759 (41%)
Outer Regional Australia	7566 (55%)	6093 (45%)
Remote Australia	5372 (49%)	5675 (51%)
Very Remote Australia	1237 (53%)	1077 (47%)
Area–level disadvantage (SEIFA)		
Quintile 1 (most disadvantage)	10 023 (50%)	9830 (50%)
Quintile 2	6839 (57%)	5148 (43%)
Quintile 3	5994 (60%)	3930 (40%)
Quintile 4	3485 (61%)	2247 (39%)
Quintile 5 (least disadvantage)	1266 (69%)	579 (31%)

	Median (range)	Median (range)
Population size	68 691 (3705, 219 929)	58 515 (1523, 219 929)
Median population		
Age	36.9 years (27.4, 54.7)	36.8 years (27.4, 54.7)
Weekly income	$635 (299, 1675)	$620 (299, 1675)
Proportion of population		
Participating in employment	59% (40, 79)	58% (38, 79)
Completed year 12	40% (24, 80)	38% (24, 80)
With private health insurance	29% (7, 57)	28% (7, 57)

^a^ No smoking self‐reported in the first 20 weeks of pregnancy.

^b^ Smoking self‐reported in the first 20 weeks of pregnancy.

### 3.2 Factors Associated With Not Smoking

The tests of spatial autocorrelation between SA3s were statistically significant (Moran's I = 0.24, *p* = < 0.001 and from the CAR multivariable model *ρ* = 0.16, *p* = 0.010), indicating that not smoking rates are positively correlated with rates in neighbouring SA3 areas. Table 2 presents the spatial regression results accounting for this autocorrelation. Most of the characteristics showed some association with not smoking in crude analyses. Multivariable CAR spatial regression 1 was run with area‐level disadvantage (SEIFA) and without its correlated factors, and regression 2 excluded SEIFA and included these factors. In these regressions, controlling for other variables, only median age, SEIFA, and employment participation rate showed a statistically significant association with not smoking (*p* < 0.05). A 1‐year increase in the median age of an SA3 was associated with a 2%–4% increase in the odds of not smoking (OR: 1.02; 95% CI: 1–1.03 for regression 1 and OR: 1.04; 95% CI: 1.02–1.06 for regression 2). Increasing SEIFA quintiles from most (quintile 1) to least disadvantaged (quintile 5) was associated with higher odds of not smoking. Areas in SEIFA quintile 2 were associated with a 31% increase in odds of not smoking compared to quintile 1 (Regression 1 OR: 1.31; 95% CI: 1.15–1.50) while areas in SEIFA quintile 5 were associated with a 147% increase in the odds of not smoking compared to quintile 1 (regression 1 OR: 2.47; 95% CI: 1.84–3.32). An increase of 1 percentage point in employment rate was associated with a 3% increase in the odds of not smoking (Regression 2 OR: 1.03; 95% CI: 1.02–1.05).

**TABLE 2:** Crude and multivariable CAR spatial regression model results for the area‐level factors associated with not smoking during pregnancy for Aboriginal and Torres Strait Islander women (*N* = 49 341).

Variable	Crude			Multivariate regression 1		Multivariate regression 2	
	OR (95% CI)	*p*	Rho	OR (95% CI)	*p*	OR (95% CI)	*p*
Total population (10000)	1.01 (0.99, 1.02)	0.319	0.159	1.01 (0.99, 1.02)	0.331	1.00 (0.99, 1.01)	0.725
Median age (1 year)	1.01 (1.00, 1.02)	0.115	0.16	1.02 (1.00, 1.03)	0.005**	1.04 (1.02, 1.06)	< 0.001***
Private Health Insurance (1%)	1.02 (1.02, 1.03)	< 0.001***	0.161	0.99 (0.98, 1.00)	0.277	1.00 (0.99, 1.01)	0.744
Remoteness (ARIA)^a^							
Major Cities of Australia	ref	0.001**	0.151	ref	0.261	ref	0.735
Inner Regional Australia	0.98 (0.86, 1.12)			1.02 (0.89, 1.16)		1.03 (0.90, 1.19)	
Outer Regional Australia	0.85 (0.72, 1.01)			0.94 (0.80, 1.10)		0.95 (0.80, 1.13)	
Remote Australia	0.58 (0.45, 0.75)			0.77 (0.60, 0.99)		0.93 (0.70, 1.23)	
Very Remote Australia	0.80 (0.63, 1.02)			0.86 (0.69, 1.09)		0.89 (0.70, 1.14)	
Area–level disadvantage (SEIFA)^b^							
Quintile 1 (most disadvantage)	ref	< 0.001***	0.159	ref	< 0.001***		
Quintile 2	1.30 (1.15, 1.47)			1.31 (1.15, 1.50)			
Quintile 3	1.33 (1.17, 1.51)			1.38 (1.18, 1.62)			
Quintile 4	1.48 (1.29, 1.71)			1.59 (1.30, 1.94)			
Quintile 5 (least disadvantage)	2.20 (1.85, 2.63)			2.47 (1.84, 3.32)			
Median weekly income ($100)	1.08 (1.05, 1.11)	< 0.001***	0.16			0.97 (0.93, 1.02)	0.267
Employment participation rate (1%)	1.02 (1.01, 1.03)	< 0.001***	0.159			1.03 (1.02, 1.05)	< 0.001***
Completed year 12 (1%)	1.01 (1.01, 1.02)	< 0.001***	0.154			1.01 (1.00, 1.02)	0.085

^a^ ARIA: Accessibility/remoteness index of Australia.

^b^ SEFIA: Socio‐economic indexes for areas.

** *p* < 0.01.

*** *p* < 0.001.

### 3.3 Geographic Variation

**FIGURE 1:**
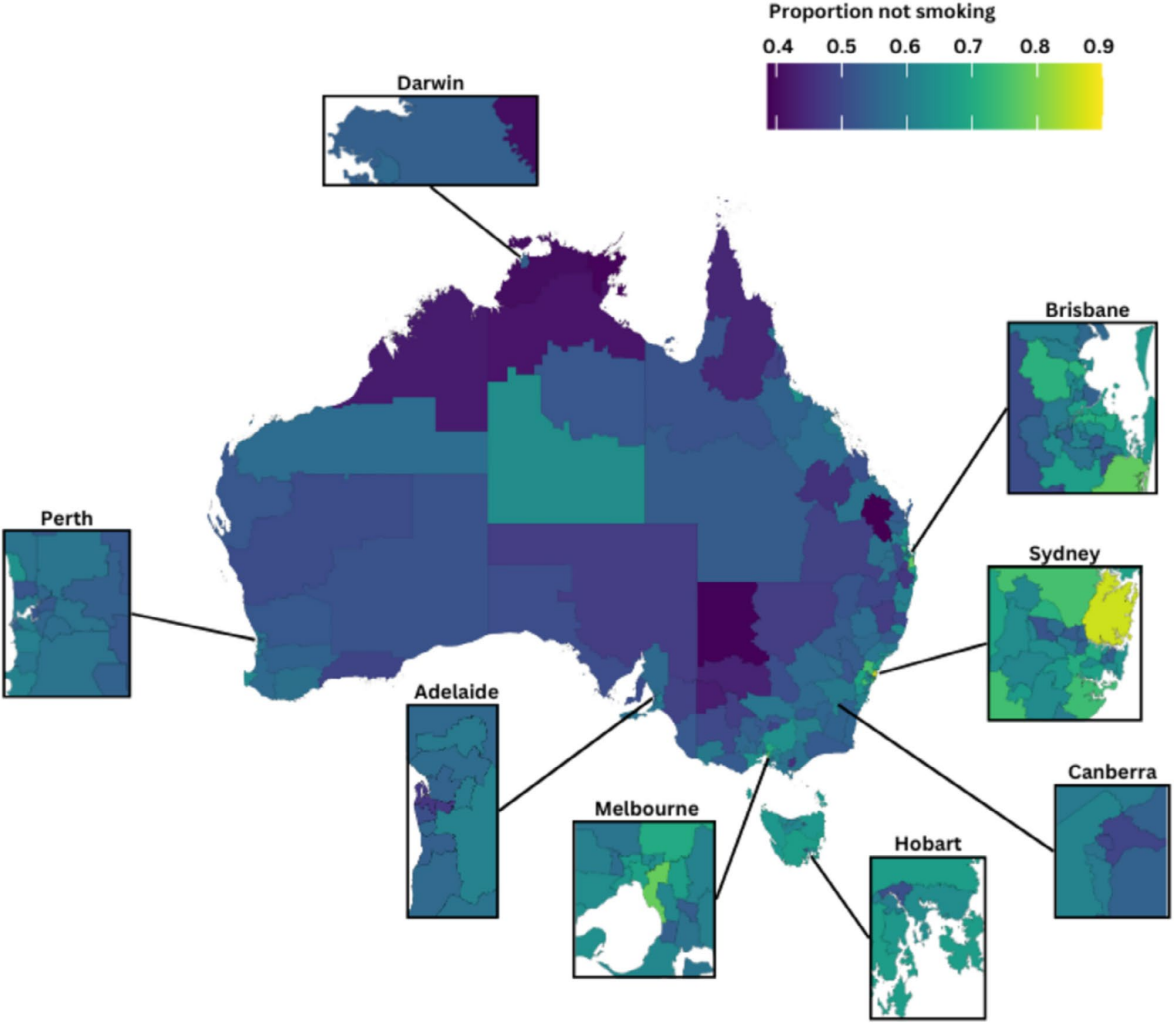
Heat map of the proportion of Aboriginal and Torres Strait Islander women not smoking during pregnancy (fitted values) by area (SA3) of usual residence (*N* = 49 341).

Figure 1 presents the regression 1 model estimate of rates of not smoking as a heat map (See Data S1 for the full list of model estimates). Combining some SA3s due to low numbers of women resulted in 239 areas being mapped. The prevalence of not smoking ranged from 39% to 86% across all areas. The highest rate of not smoking (86%) was found in a combined cluster of 9 SA3s in the northern suburbs of Sydney, NSW (3 SA4s: North Sydney and Hornsby, Northern Beaches and Ryde). A further 2 regions had prevalences of not smoking during the first 20 weeks of pregnancy above 75%, including suburbs in the Inner East and Inner South of Melbourne, VIC (2 SA4s consisting of a total of 7 SA3s) and the Gold Coast region of QLD (SA4 consisting of 10 SA3s). Three SA3s had a prevalence of not smoking below 40%: Burnett, QLD; Broken Hill and Far West, NSW; and East Arnhem, NT.

## 4 Discussion

The study found that smoking during pregnancy among Aboriginal and Torres Strait Islander women was strongly associated with area‐level socioeconomic disadvantage. Those living in less disadvantaged areas were more likely not to smoke during pregnancy than those living in more disadvantaged areas. The measure of disadvantage used in this study, SEIFA, is a composite measure that incorporates variables associated with household income, education, and employment, as well as other factors [32]. Analysis of these individual components found that employment was the main driver of this finding. That is, women living in areas with high levels of participation in paid employment were more likely not to smoke in the first 20 weeks of pregnancy than women in areas of low employment.

Previous research has found that smoking prevalence is higher among disadvantaged groups, including disadvantaged pregnant women [33]. Several factors may contribute to this trend, including increased stress due to social, emotional and financial pressures, a lack of motivation to quit and targeting by tobacco advertising [33, 34, 35, 36]. Although socioeconomic factors are widely recognised as key drivers of smoking behaviour, particularly during pregnancy, for Aboriginal and Torres Strait Islander peoples, these factors are directly shaped by the ongoing impacts of colonisation, including systemic discrimination, disconnection from Country and culture, intergenerational trauma and the structural exclusion from employment and education opportunities [37]. These structural inequities underpin high rates of smoking among pregnant Aboriginal and Torres Strait Islander women and must be acknowledged when interpreting the link between the area‐level factors examined as part of this study.

In the current study, the employment rate of the geographic area of a woman's usual place of residence was associated with her smoking behaviours. Residing in an area with stronger employment prospects may lead to more optimistic views about the future, positively influencing maternal behaviour during pregnancy [38]. Women who are employed may have better access to support systems and resources that encourage not smoking. In areas with high employment, the prevalence of non‐smoking partners and peers is also likely to be higher, resulting in higher levels of social support and fewer opportunities to smoke [39, 40]. This social support can be particularly important for women who might otherwise feel isolated or stressed, mitigating smoking being used as a coping mechanism [41]. Employment can also provide a sense of stability, structure, and purpose that supports healthier habits, reducing opportunities or desires to smoke [42].

Remoteness is often identified as a factor associated with smoking [6, 43]. Although this study found that pregnant Aboriginal and Torres Strait Islander women living in major cities had a higher prevalence of not smoking than those living in more regional and remote areas, this was not significant in multivariate regressions. This finding suggests that remoteness alone is not a driver of smoking behaviour during pregnancy among Aboriginal and Torres Strait Islander women in the time period examined. In Australia, regional and remote areas typically face challenges to good health, including less access to health and wellbeing services, lower rates of education and employment, and greater socioeconomic disadvantage [44]. The findings of this study suggest that enhancing employment opportunities and addressing barriers to employment in these regions may be key points for interventions to contribute to reducing maternal smoking rates. In addition, future research should explore integrating culturally appropriate health promotion messaging and quit support into workplaces as part of broader efforts to support smoke‐free pregnancies.

Identifying areas with lower non‐smoking rates is critical to inform the targeted provision of smoking prevention and cessation services. To maximise the effectiveness of targeted smoking cessation efforts, it is also essential to understand smoking behaviours within particular areas and contexts. For example, the current study indicates that Alice Springs (in the Northern Territory) has a low rate of smoking; however, the practice of chewing tobacco (pituri) is common in this area and often replaces smoking of tobacco [10, 45]. Although the use of pituri during pregnancy is not well studied, there is emerging evidence that it may be linked to elevated glucose and diabetes [45]. This highlights the importance of understanding regional variations in not only prevalence but also types of tobacco use. In this context, the Australian government funded Indigenous‐led Tackling Indigenous Smoking (TIS) program is playing a critical role in delivering place‐based and locally responsive health promotion approaches that promote smoking cessation among Aboriginal and Torres Strait Islander people, including pregnant women [46, 47]. Our findings provide important data that may be useful in strengthening and tailoring TIS activities to areas of high smoking prevalence. Leveraging the community‐led nature of the TIS teams and regional knowledge can enhance cultural and place‐based prevalence of smoking cessation approaches.

## 5 Strengths and Limitations

This study took a strengths‐based approach by investigating factors associated with not smoking that can be used when developing policies and initiatives. The study used data from the NPDC, a nationally collected dataset and therefore had good coverage from all areas of Australia. Some areas, however, had low numbers of births and needed to be combined with other areas to maintain individuals' privacy. Since the data is deidentified, there is no way to identify women who had multiple births in the study time period, so they are included multiple times. The dataset relies on women accurately responding to questions about smoking and could therefore overestimate the true rate of not smoking. It is possible that differences between jurisdictions and services will result in these questions being asked or recorded differently in different areas; however, established indicator definitions and guidelines and validation of the data aim to reduce this [48]. Care should be taken in understanding that area‐level factors may not accurately reflect the circumstances of all pregnant women within that area. In addition, our analysis only includes Aboriginal and Torres Strait Islander women, not all mothers of Aboriginal and Torres Strait Islander babies, as this is how national data is reported [6]. Therefore, the findings should be interpreted within this context. Although smoking rates have continued to decrease since the data used in the study was collected (from 49% in 2011 to 40% in 2022 [49]), the results remain relevant as the structural and systemic factors persist.

## 6 Conclusions

Geographic variation in rates of not smoking during the first 20 weeks of pregnancy is associated with socioeconomic disadvantage. This is primarily driven by employment participation rates, with women living in areas of high employment less likely to smoke during pregnancy than women living in areas of low employment. Resources to address smoking in pregnancy can be directed to areas identified as having high rates of maternal smoking and should consider the inclusion of strategies that enhance employment opportunities.

## Ethics Statement

Ethics approvals were obtained from the Aboriginal Health and Medical Research Council (1588/19), Aboriginal Health Research Ethics Committee SA (04‐19‐853), ACT Health Human Research Ethics Committee (2019.ETH.00231), Human Research Ethics Committee of Northern Territory Health and Menzies School of Health Research (2019‐3580), QLD Public Health Act (EO2020/1/1134), SA Health HREC, University of Newcastle's Human Research Ethics Committee (H‐2019‐0414), and the Australian Institute of Health and Welfare Ethics Committee (EO2020/1/1134). The research was conducted in line with the National Health and Medical Research Council's Guidelines for ethical conduct in Aboriginal and Torres Strait Islander health research, the Aboriginal Health and Medical Research Council's ethical guidelines: key principles (2020) V2.0 and the international CONSIDER statement.

## Conflicts of Interest

The authors declare no conflicts of interest.

## Supporting information


**Data S1:** Proportion (fitted from model) of pregnant Aboriginal and Torres Strait Islander women who reported not smoking in the first 20 weeks of pregnancy in each SA3 location in Australia (areas with low numbers were combined for analysis as indicated).

## Data Availability

Access to the National Perinatal Data Collection used in this study can be arranged through the Australian Institute of Health and Welfare.
