# Supplementary material for: Factors Contributing to Geographical Variation in Maternal Smoking Rates Among Aboriginal and Torres Strait Islander Women
Source: Health Promot J Austr. 2025 Sep 7;36(4):e70095. doi: 10.1002/hpja.70095 (PMC12415412; doi:10.1002/hpja.70095)
Supplement: Supplementary file 1 — Data S1: Proportion (fitted from model) of pregnant Aboriginal and Torres Strait Islander women who reported not smoking in the first 20 weeks of pregnancy in each SA3 location in Australia (areas with low numbers were combined for analysis as indicated). [file HPJA-36-0-s001.docx]

***Supporting Information***

**File 1:** Proportion (fitted from model) of pregnant Aboriginal and Torres Strait Islander women who reported not smoking in the first 20 weeks of pregnancy in each SA3 location in Australia (areas with low numbers were combined for analysis as indicated).

| **Statistical Area**  **(SA3)** | **State** | **Location** | **Proportion (fitted)**  **not smoking** |
| --- | --- | --- | --- |
| 10101 | NSW | Goulburn – Yass | 0.59 |
| 10102, 10103 | NSW | Queanbeyan, Snowy Mountains | 0.54 |
| 10104 | NSW | South Coast | 0.55 |
| 10201 | NSW | Gosford | 0.68 |
| 10202 | NSW | Wyong | 0.67 |
| 10301 | NSW | Bathurst | 0.53 |
| 10302 | NSW | Lachlan Valley | 0.56 |
| 10303 | NSW | Lithgow - Mudgee | 0.54 |
| 10304 | NSW | Orange | 0.58 |
| 10401 | NSW | Clarence Valley | 0.60 |
| 10402 | NSW | Coffs Harbour | 0.61 |
| 10501 | NSW | Bourke - Cobar - Coonamble | 0.48 |
| 10502 | NSW | Broken Hill and Far West | 0.39 |
| 10503 | NSW | Dubbo | 0.54 |
| 10601 | NSW | Lower Hunter | 0.59 |
| 10602 | NSW | Maitland | 0.66 |
| 10603 | NSW | Port Stephens | 0.63 |
| 10604 | NSW | Upper Hunter | 0.56 |
| 10701, 10702 | NSW | Dapto - Port Kembla, | 0.59 |
| 10703 | NSW | Kiama - Shellharbour | 0.62 |
| 10704 | NSW | Wollongong | 0.60 |
| 10801 | NSW | Great Lakes | 0.60 |
| 10802 | NSW | Kempsey - Nambucca | 0.50 |
| 10804 | NSW | Port Macquarie | 0.63 |
| 10805 | NSW | Taree - Gloucester | 0.54 |
| 10901 | NSW | Albury | 0.54 |
| 10902 | NSW | Lower Murray | 0.44 |
| 10903 | NSW | Upper Murray exc. Albury | 0.59 |
| 11001 | NSW | Armidale | 0.48 |
| 11002 | NSW | Inverell - Tenterfield | 0.50 |
| 11003 | NSW | Moree - Narrabri | 0.52 |
| 11004 | NSW | Tamworth - Gunnedah | 0.57 |
| 11101 | NSW | Lake Macquarie - East | 0.68 |
| 11102 | NSW | Lake Macquarie - West | 0.68 |
| 11103 | NSW | Newcastle | 0.62 |
| 11201 | NSW | Richmond Valley - Coastal | 0.65 |
| 11202 | NSW | Richmond Valley - Hinterland | 0.47 |
| 11203 | NSW | Tweed Valley | 0.68 |
| 11301 | NSW | Griffith - Murrumbidgee (West) | 0.51 |
| 11302 | NSW | Tumut - Tumbarumba | 0.55 |
| 11303 | NSW | Wagga Wagga | 0.56 |
| 11401, 90103 | NSW | Shoalhaven, Jervis Bay | 0.61 |
| 11402 | NSW | Southern Highlands | 0.65 |
| 11501, 11502, 11503, 11504 | NSW | Baulkham Hills, Dural - Wisemans Ferry, Hawkesbury, Rouse Hill - McGraths Hill | 0.75 |
| 11601 | NSW | Blacktown | 0.57 |
| 11602 | NSW | Blacktown - North | 0.71 |
| 11603 | NSW | Mount Druitt | 0.52 |
| 11701 | NSW | Botany | 0.63 |
| 11702 | NSW | Marrickville - Sydenham - Petersham | 0.56 |
| 11703 | NSW | Sydney Inner City | 0.54 |
| 11801, 11802 | NSW | Eastern Suburbs - North, Eastern Suburbs - South | 0.65 |
| 11901 | NSW | Bankstown | 0.70 |
| 11902 | NSW | Canterbury | 0.63 |
| 11903, 11904 | NSW | Hurstville, Kogarah-Rockdale | 0.68 |
| 12002 | NSW | Leichhardt | 0.65 |
| 12001, 12003 | NSW | Canada Bay, Strathfield - Burwood - Ashfield | 0.71 |
| 12101, 12102, 12103, 12104, 12201, 12202, 12203, 12601, 12602 | NSW | Chatswood - Lane Cove, Hornsby, Ku-ring-gai, North Sydney - Mosman, Manly, Pittwater, Warringah, Pennant Hills - Epping, Ryde - Hunters Hill | 0.86 |
| 12301 | NSW | Camden | 0.67 |
| 12302 | NSW | Campbelltown (NSW) | 0.63 |
| 12303 | NSW | Wollondilly | 0.75 |
| 12401, 12402 | NSW | Blue Mountains, Penrith | 0.69 |
| 12403 | NSW | Penrith | 0.63 |
| 12404 | NSW | Richmond - Windsor | 0.62 |
| 12405 | NSW | St Marys | 0.62 |
| 12501 | NSW | Auburn | 0.55 |
| 12502 | NSW | Carlingford | 0.55 |
| 12503 | NSW | Merrylands - Guildford | 0.61 |
| 12504 | NSW | Parramatta | 0.54 |
| 12701 | NSW | Bringelly - Green Valley | 0.62 |
| 12702 | NSW | Fairfield | 0.64 |
| 12703 | NSW | Liverpool | 0.66 |
| 12801, 12802 | NSW | Cronulla - Miranda - Caringbah, Sutherland - Menai - Heathcote | 0.74 |
| 20101 | VIC | Ballarat | 0.58 |
| 20102, 20103 | VIC | Creswick - Daylesford - Ballan, Maryborough - Pyrenees | 0.56 |
| 20202, 20203 | VIC | Heathcote - Castlemaine - Kyneton, Loddon - Elmore | 0.54 |
| 20301, 20302, 20303 | VIC | Barwon - West, Geelong, Surf Coast - Bellarine Peninsula | 0.64 |
| 20401, 20402 | VIC | Upper Goulburn Valley, Wangaratta - Benalla | 0.64 |
| 20403 | VIC | Wodonga - Alpine | 0.61 |
| 20501 | VIC | Baw Baw | 0.58 |
| 20502 | VIC | Gippsland - East | 0.54 |
| 20504 | VIC | Latrobe Valley | 0.46 |
| 20503, 20505 | VIC | Gippsland - South West, Wellington | 0.58 |
| 20601, 20602, 20603, 20604, 20605, 20606, 20607 | VIC | Brunswick - Coburg, Darebin - South, Essendon, Melbourne City, Port Phillip, Stonnington - West, Yarra | 0.68 |
| 20701, 20702, 20703, 20801, 20802, 20803, 20804 | VIC | Boroondara, Manningham - West, Whitehorse - West, Bayside, Glen Eira, Kingston, Stonnington - East | 0.78 |
| 20901 | VIC | Banyule | 0.64 |
| 20902 | VIC | Darebin - North | 0.63 |
| 20903, 20904 | VIC | Nillumbik - Kinglake, Whittlesea - Wallan | 0.70 |
| 21001, 21003 | VIC | Keilor, Moreland - North | 0.66 |
| 21002, 21004, 21005 | VIC | Macedon Ranges, Sunbury, Tullamarine - Broadmeadows | 0.59 |
| 21101, 21102, 21103, 21104 | VIC | Knox, Manningham - East, Maroondah, Whitehorse - East | 0.67 |
| 21105 | VIC | Yarra Ranges | 0.61 |
| 21201 | VIC | Cardinia | 0.61 |
| 21202 | VIC | Casey - North | 0.54 |
| 21203 | VIC | Casey - South | 0.58 |
| 21204, 21205 | VIC | Dandenong, Monash | 0.62 |
| 21301 | VIC | Brimbank | 0.57 |
| 21302, 21303 | VIC | Hobsons Bay, Maribyrnong | 0.65 |
| 21304 | VIC | Melton - Bacchus Marsh | 0.61 |
| 21305 | VIC | Wyndham | 0.63 |
| 21401 | VIC | Frankston | 0.54 |
| 21402 | VIC | Mornington Peninsula | 0.62 |
| 21501 | VIC | Grampians | 0.50 |
| 21502 | VIC | Mildura | 0.43 |
| 21503 | VIC | Murray River - Swan Hill | 0.49 |
| 21601, 21602 | VIC | Campaspe, Moira | 0.53 |
| 21603 | VIC | Shepparton | 0.49 |
| 21701 | VIC | Glenelg - Southern Grampians | 0.59 |
| 21702 | VIC | Warrnambool – Otway Ranges | 0.53 |
| 30101 | QLD | Capalaba | 0.70 |
| 30102 | QLD | Cleveland - Stradbroke | 0.66 |
| 30103 | QLD | Wynnum - Manly | 0.67 |
| 30201 | QLD | Bald Hills - Everton Park | 0.63 |
| 30202 | QLD | Chermside | 0.58 |
| 30203 | QLD | Nundah | 0.55 |
| 30204 | QLD | Sandgate | 0.63 |
| 30301, 30302 | QLD | Carindale and Holland park, Cleveland - Stradbroke | 0.71 |
| 30303 | QLD | Mt Gravatt | 0.64 |
| 30304 | QLD | Nathan | 0.62 |
| 30305 | QLD | Rocklea - Acacia Ridge | 0.61 |
| 30306 | QLD | Sunnybank | 0.58 |
| 30401, 30402, 30403, 30404 | QLD | Centenary, Kenmore - Brookfield - Moggill, Sherwood - Indooroopilly, The Gap - Enoggera | 0.60 |
| 30501, 30502, 30503, 30504 | QLD | Brisbane Inner, Brisbane Inner - East, Brisbane Inner - North, Brisbane Inner - West | 0.67 |
| 30601 | QLD | Cairns - North | 0.64 |
| 30602 | QLD | Cairns - South | 0.55 |
| 30603 | QLD | Innisfail - Cassowary Coast | 0.49 |
| 30604 | QLD | Port Douglas - Daintree | 0.58 |
| 30605 | QLD | Tablelands (East) - Kuranda | 0.49 |
| 30701 | QLD | Darling Downs (West) - Maranoa | 0.49 |
| 30702 | QLD | Darling Downs - East | 0.57 |
| 30703 | QLD | Granite Belt | 0.57 |
| 30801 | QLD | Central Highlands (Qld) | 0.46 |
| 30802 | QLD | Gladstone – Biloela | 0.58 |
| 30803 | QLD | Rockhampton | 0.51 |
| 30901, 30902, 30903, 30904, 30905, 30906, 30907, 30908, 30909, 30910 | QLD | Broadbeach - Burleigh, Coolangatta, Gold Coast - North, Gold Coast Hinterland, Mudgeeraba - Tallebudgera, Nerang, Ormeau - Oxenford, Robina, Southport, Surfers Paradise | 0.78 |
| 31001 | QLD | Forest Lake - Oxley | 0.54 |
| 31002 | QLD | Ipswich Hinterland | 0.51 |
| 31003 | QLD | Ipswich Inner | 0.55 |
| 31004 | QLD | Springfield - Redbank | 0.64 |
| 31101 | QLD | Beaudesert | 0.47 |
| 31102 | QLD | Beenleigh | 0.51 |
| 31103 | QLD | Browns Plains | 0.58 |
| 31104 | QLD | Jimboomba | 0.67 |
| 31105 | QLD | Loganlea - Carbrook | 0.59 |
| 31106 | QLD | Springwood - Kingston | 0.53 |
| 31201 | QLD | Bowen Basin - North | 0.57 |
| 31202 | QLD | Mackay | 0.60 |
| 31203 | QLD | Whitsunday | 0.58 |
| 31301 | QLD | Bribie - Beachmere | 0.56 |
| 31302, 31303 | QLD | Caboolture, Caboolture Hinterland | 0.60 |
| 31304 | QLD | Narangba - Burpengary | 0.61 |
| 31305 | QLD | Redcliffe | 0.63 |
| 31401 | QLD | The Hills District | 0.72 |
| 31402 | QLD | North Lakes | 0.71 |
| 31403 | QLD | Strathpine | 0.63 |
| 31501 | QLD | Far North | 0.44 |
| 31502 | QLD | Outback - North | 0.52 |
| 31503 | QLD | Outback - South | 0.54 |
| 31602 | QLD | Caloundra | 0.63 |
| 31603, 31605 | QLD | Maroochy, Noosa | 0.64 |
| 31601, 31604, 31606 | QLD | Buderim, Nambour – Pomona, Sunshine Coast Hinterland | 0.66 |
| 31701 | QLD | Toowoomba | 0.58 |
| 31801 | QLD | Charters Towers - Ayr - Ingham | 0.55 |
| 31802 | QLD | Townsville | 0.64 |
| 31901 | QLD | Bundaberg | 0.54 |
| 31902 | QLD | Burnett | 0.39 |
| 31903 | QLD | Gympie - Cooloola | 0.55 |
| 31904 | QLD | Hervey Bay | 0.55 |
| 31905 | QLD | Maryborough | 0.53 |
| 40101, 40102, 40103, 40104, 40105, 40106, 40107 | SA | Adelaide City, Adelaide Hills, Burnside, Campbelltown (SA), Norwood - Payneham - St Peters, Prospect - Walkerville, Unley | 0.61 |
| 40201 | SA | Gawler - Two Wells | 0.59 |
| 40202 | SA | Playford | 0.56 |
| 40203 | SA | Port Adelaide - East | 0.47 |
| 40204 | SA | Salisbury | 0.55 |
| 40205 | SA | Tea Tree Gully | 0.60 |
| 40301, 40302, 40303 | SA | Holdfast Bay, Marion, Mitcham | 0.54 |
| 40304 | SA | Onkaparinga | 0.56 |
| 40401 | SA | Charles Sturt | 0.51 |
| 40402 | SA | Port Adelaide - West | 0.47 |
| 40403 | SA | West Torrens | 0.56 |
| 40501, 40502, 40503 | SA | Barossa, Lower North, Mid North | 0.56 |
| 40504 | SA | Yorke Peninsula | 0.50 |
| 40601 | SA | Eyre Peninsula and South West | 0.51 |
| 40602 | SA | Outback - North and East | 0.48 |
| 40701 | SA | Fleurieu - Kangaroo Island | 0.58 |
| 40702 | SA | Limestone Coast | 0.53 |
| 40703 | SA | Murray and Mallee | 0.49 |
| 50101 | WA | Augusta - Margaret River - Busselton | 0.63 |
| 50102, 50103 | WA | Bunbury, Manjimup | 0.56 |
| 50201 | WA | Mandurah | 0.59 |
| 50301, 50302 | WA | Cottesloe - Claremont, Perth City | 0.60 |
| 50401 | WA | Bayswater - Bassendean | 0.56 |
| 50402 | WA | Mundaring | 0.54 |
| 50403 | WA | Swan | 0.59 |
| 50501 | WA | Joondalup | 0.65 |
| 50502 | WA | Stirling | 0.52 |
| 50503 | WA | Wanneroo | 0.58 |
| 50601, 50606 | WA | Armadale, Serpentine - Jarrahdale | 0.58 |
| 50602 | WA | Belmont - Victoria Park | 0.53 |
| 50603 | WA | Canning | 0.56 |
| 50604 | WA | Gosnells | 0.57 |
| 50605 | WA | Kalamunda | 0.58 |
| 50607 | WA | South Perth | 0.52 |
| 50701 | WA | Cockburn | 0.61 |
| 50702 | WA | Fremantle | 0.54 |
| 50703 | WA | Kwinana | 0.59 |
| 50704 | WA | Melville | 0.60 |
| 50705 | WA | Rockingham | 0.61 |
| 50806 | WA | Pilbara | 0.57 |
| 50901 | WA | Albany | 0.57 |
| 50902 | WA | Wheat Belt - North | 0.53 |
| 50903 | WA | Wheat Belt - South | 0.55 |
| 51001 | WA | Kimberley | 0.42 |
| 51101 | WA | Esperance | 0.49 |
| 51102 | WA | Gascoyne | 0.53 |
| 51103 | WA | Goldfields | 0.52 |
| 51104 | WA | Mid West | 0.51 |
| 60101 | TAS | Brighton | 0.51 |
| 60102, 60106 | TAS | Hobart - North East, Sorell - Dodges Ferry | 0.63 |
| 60103 | TAS | Hobart - North West | 0.60 |
| 60104, 60105 | TAS | Hobart - South and West, Hobart Inner | 0.66 |
| 60201 | TAS | Launceston | 0.55 |
| 60202 | TAS | Meander Valley - West Tamar | 0.62 |
| 60203 | TAS | North East | 0.66 |
| 603 | TAS | South East | 0.67 |
| 60401 | TAS | Burnie - Ulverstone | 0.63 |
| 60402 | TAS | Devonport | 0.61 |
| 60403 | TAS | West Coast | 0.66 |
| 70101, 70102, 70103 | NT | Darwin City, Darwin Suburbs, Litchfield | 0.54 |
| 70104 | NT | Palmerston | 0.56 |
| 70201 | NT | Alice Springs | 0.64 |
| 70202 | NT | Barkly | 0.52 |
| 70203 | NT | Daly - Tiwi - West Arnhem | 0.41 |
| 70204 | NT | East Arnhem | 0.39 |
| 70205 | NT | Katherine | 0.41 |
| 80101, 80102, 80104 | ACT | Belconnen, Cotter- Namadgi, Gungahlin | 0.62 |
| 80103, 80105 | ACT | Canberra East, North Canberra | 0.49 |
| 80106 | ACT | South Canberra | 0.50 |
| 80107 | ACT | Tuggeranong | 0.59 |
| 80108, 80109 | ACT | Weston Creek, Woden Valley | 0.58 |
